# Impact of ambulatory palliative care on symptoms and service outcomes in cancer patients: a retrospective cohort study

**DOI:** 10.1186/s12904-022-00924-5

**Published:** 2022-03-04

**Authors:** Rajvi Shah, Ekavi N. Georgousopoulou, Ziad Al-Rubaie, Merlina Sulistio, Hoong Tee, Adelaide Melia, Natasha Michael

**Affiliations:** 1 Supportive, Psychosocial and Palliative Care Research Department, Cabrini Health, 154 Wattletree Road, Malvern, VIC 3144 Australia; 2 School of Medicine, Sydney Campus, University of Notre Dame Australia, Sydney, NSW Australia; 3 Faculty of Medicine, Nursing and Health Sciences, Monash University, Melbourne, VIC Australia

**Keywords:** Cancer pain, Cancer symptom, Outpatient, Ambulatory, Palliative care, End-of-life care, Clinic

## Abstract

**Background:**

The integration of palliative care into routine cancer care has allowed for improved symptom control, relationship building and goal setting for patients and families. This study aimed to assess the efficacy of an ambulatory palliative care clinic on improving symptom burden and service outcomes for patients with cancer.

**Methods:**

A retrospective review of data of cancer patients who attended an ambulatory care clinic and completed the Symptom Assessment Scale between January 2015 and December 2019. We classified moderate to severe symptoms as clinically significant. Clinically meaningful improvement in symptoms (excluding pain) was defined by a ≥ 1-point reduction from baseline and pain treatment response was defined as a ≥ 2-point or ≥ 30% reduction from baseline.

**Results:**

A total of 249 patients met the inclusion criteria. The most common cancer diagnosis was gastrointestinal (32%) and the median time between the initial and follow-up clinic was 4 weeks. The prevalence of clinically significant symptoms at baseline varied from 28% for nausea to 88% for fatigue, with 23% of the cohort requiring acute admission due to unstable physical/psychosocial symptoms. There was significant improvement noted in sleep (*p* < 0.001), pain (*p* = 0.002), wellbeing (*p* < 0.001), and overall symptom composite scores (*p* = 0.028). Despite 18–28% of patients achieving clinically meaningful symptom improvement, 18–66.3% of those with moderate to severe symptoms at baseline continued to have clinically significant symptoms on follow-up. A third of patients had opioid and/or adjuvant analgesic initiated/titrated, with 39% educated on pain management. Goals of care (31%), insight (28%) and psychosocial/existential issues (27%) were commonly explored.

**Conclusions:**

This study highlights the burden of symptoms in a cohort of ambulatory palliative care patients and the opportunity such services can provide for education, psychosocial care and future planning. Additionally routine screening of cohorts of oncology patients using validated scales may identify patients who would benefit from early ambulatory palliative care.

## Introduction

Despite advances in cancer treatment and its subsequent impact on prognosis and trajectory, unrelieved symptoms continue to have a debilitating effect on patients and families, evoking much suffering and impeding the attainment of an acceptable quality of life (QOL) [1, 2]. Cancer symptoms commonly present as clusters, vacillate in their severity and may be attributed to disease or treatment and may continue into survivorship [3, 4]. Symptoms include, but are not limited to, pain, anorexia, fatigue, shortness of breath, nausea, constipation, and anxiety, with a prevalence ranging from 50 to 84% [5, 6].

The emergence of palliative care as a mainstream specialty and integration of early palliative care into cancer care has allowed for the early identification, assessment and treatment of pain and other symptoms [7]. Multiple international studies have highlighted that early palliative care, when provided alongside standard oncological care, leads to improved patient outcomes and clinically meaningful improvements in QOL and survival [8]. The expansion of cancer care in the ambulatory setting has supported the concurrent development of ambulatory palliative care clinics [9, 10]. Such clinics allow for the collaborative integration of palliative care into routine cancer care to assist with symptom control, relationship building, continuity of care and goal setting [11, 12]. Guidelines in Australia suggest that ambulatory palliative care services should be provided in ambulatory clinics or specialist rooms but with no specific articulations for best practice in the Australian cancer setting [13].

An international Delphi study recommends 11 major criteria for referral to ambulatory palliative care: severe physical and emotional symptoms, request for hastened death, spiritual or existential crisis, assistance with decision making or care planning, patient request for a referral, delirium, spinal cord compression, brain or leptomeningeal metastases, within 3 months of an advanced cancer diagnosis for patients with a median survival of 1 year or less, and progressive disease despite second-line therapy [14]. Additionally, ambulatory palliative care referrals should be triggered by symptom screening in conjunction with clinician-based referrals [15].

International studies assessing the efficacy of ambulatory palliative care on symptom burden and patient satisfaction with care commonly focused on measurable changes in patient-reported outcomes measured through multi-item symptom assessment scales [16, 17]. A commonly used scale, the Edmonton Symptom Assessment Scale (ESAS), measures 11 common cancer symptoms on a numerical rating scale (NRS) [18, 19]. A phase II Canadian study of 88 cancer patients attending ambulatory palliative care demonstrated significant improvements in pain, fatigue, nausea, anxiety, dyspnoea and insomnia (*p* ≤ 0.0001), as well as depression, drowsiness, and constipation (*p* ≤ 0.002) at 1-month follow-up [11]. A subsequent retrospective longitudinal study in the United States of 1612 cancer patients with moderate to severe symptoms at baseline (NRS ≥ 4) demonstrated statistically significant improvement in all ESAS symptom domains between the initial review and subsequent follow up (*p* < 0.001), with 45% (*p* < 0.001) demonstrating a two-point difference or 30% reduction in pain intensity on NRS [4, 20]. A further study of 182 patients with stage IV cancer reviewed in an ambulatory palliative care clinic in Jordan found improvement in pain (*p* = 0.004) and sleep disturbance (*p* = 0.007) regardless of baseline symptom intensity score, with statistically significant improvement in all ESAS domains for patients with moderate to severe symptoms (NRS ≥ 4) (*p* ≤ 0.003) [5].

As part of our organisation’s integrated specialist palliative care service (inpatient, community, consult and ambulatory), an ambulatory palliative care clinic, known as the Supportive Care Clinic (SCC), was established in 2015. The aim of this study was to retrospectively assess the impact of the SCC on clinical and service outcomes, including assessing for improvement in symptom burden, clinical interventions implemented and evaluating forward referrals to other aspects of the service. To our knowledge, this is the first study to assess the impact of an ambulatory palliative care clinic in an Australian setting.

## Methods

### Design

We conducted a retrospective cohort study of all new patients presenting to the SCC between January 2015 and December 2019 in a single institution. Eligible patients were at least 18 years old, diagnosed with a solid organ or haematological malignancy of any stage and were newly assessed by a Palliative Care Physician in the ambulatory setting. Patients who had incomplete medical records or missing physician correspondence from the initial appointment were excluded from this study. The study complied with the STROBE guidelines for the reporting of observational studies. Ethics was granted by the organisation’s Human Research Ethics Committee (Number: 08–18–05-20) and the study was funded by the Cabrini Foundation Quality Improvement Grant.

### Setting

This study was conducted at a large not-for-profit, private health care service in Melbourne, Australia, providing acute, sub-acute and community-based care across six campuses. Specialist Palliative Care is available via an integrated service, comprising a 22-bed inpatient unit, a hospital consultation service, a community service and ambulatory Supportive Care Clinics. The Supportive Care Clinic is staffed by a specialist accredited Palliative Medicine Physician, supported by a nurse, allied health team, pharmacist and is physically located within the Haematology and Oncology Centre. All patients who attend the SCC complete a Symptom Assessment Scale (SAS) on arrival [21].

### Data sources and outcomes

We used the hospital’s administrative database to identify patients who attended the clinic during the study period. New attendees at the clinic were identified using the Medicare Benefits Schedule item number for a first review [22]. Electronic medical records of identified patients were reviewed against the remaining eligibility criteria. We gathered information on patient demographics, presence and severity of symptoms, and clinical and service outcomes following clinic review.

To assess patient-reported symptom outcomes, we used the SAS [21]. The SAS utilises an NRS (0–10), similar to the ESAS, and is used by palliative care services across Australia involved in the Palliative Care Outcomes Collaboration [23]. The SAS assesses the amount of distress caused by symptoms across 8 domains: sleep disturbance, anorexia, nausea, bowel problems, dyspnoea, fatigue, pain, and other symptoms and demonstrates strong psychometric properties of internal consistency (α = 0.64–0.92) and reliability (*r* = 0.84–0.92) [21]. Impairments in overall mood and wellbeing/QOL were additionally assessed on an NRS (0–10). We reviewed data from the initial clinic assessment and those who attended a follow-up clinic within 8 weeks of their initial appointment.

Intensity of symptoms reported on the SAS were categorised as follows: NRS 0 = absent, 1–3 = mild, 4–7 = moderate and 8–10 = severe [21]. Clinically significant symptoms were classified as those with NRS ≥ 4, while a ≥ 1-point reduction in NRS defined a clinically meaningful improvement for all symptoms excluding pain [24]. For pain, we assessed pain treatment response, defined as ≥2-point reduction or ≥ 30% reduction from baseline pain score [25]. A symptom composite score was calculated by summing the 8 SAS items [11]. The score was prorated as long as the participant has completed more than 50% of the SAS items with prorated scores calculated by summing the individual scores, multiplying by the number of possible items (8 items), and dividing by the total number of items completed [11].

Corresponding physician clinic correspondence was examined by three investigators (HT, RS, AM). We developed a standardised data collection sheet to obtain the following data: basic demographics, medication changes and their indications, referrals generated, physical, social and psycho-existential issues explored during the consultation and patient disposition after the clinic appointment. An initial pilot study was conducted to confirm the utility and appropriateness of the data collected. A third of the data was reassessed to ensure concordance in data obtained between reviewers.

### Statistical analysis

Patients’ socio-demographic characteristics, presence and intensity of baseline symptoms, and appointment outcomes of interest were summarised using descriptive statistics, including mean, median, frequencies and valid percentages. Where patients had a second clinic appointment, we used Paired t-test (or Wilcoxon signed rank test when appropriate) to test for changes in symptom intensity scores between the two time-points. A significance criterion of *p* ≤ 0.05 was used in the analysis. Statistical analysis was performed using SPSS V.25.0 on valid data.

## Results

### Patient characteristics

**Fig. 1:**
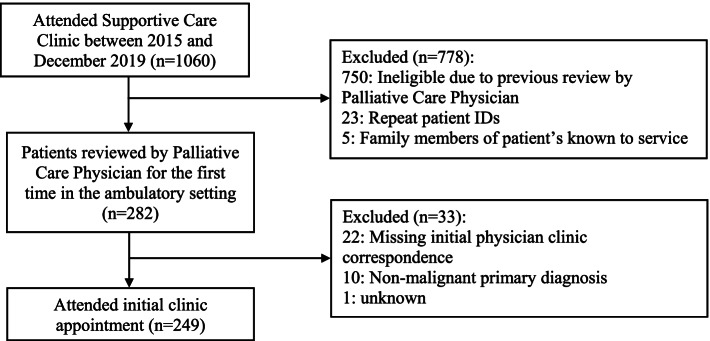
Subject selection

Of 1060 patients reviewed in the SCC over the 5 years, 282 met the eligibility criteria and data from 249 patients was evaluated (Fig. 1). The baseline characteristics of the patients are summarised in Table 1. The mean patient age at the initial clinic appointment was 68.7 years, the majority were female (53.8%), and the most common cancer diagnosis was gastrointestinal, encompassing colorectal and pancreatic cancer (*n* = 80, 32.1%). At the conclusion of the study period, 204 (81.9%) of the study cohort were deceased, and the mean age at death was 69 years. The median time between the first referral to the integrated palliative care service and initial SCC appointment was 110 days, and the first referral to the service and death was 152.5 days. Over half the patients (54.9%) died in a dedicated palliative care unit, whilst a quarter (25.5%) died at their usual community residence.

**Table 1:** Patient characteristics

	*n* = 249(%)^a^
Age at initial clinic appointment, mean in years (SD)	68.7 (12.7)
Sex	
Female	134 (53.8)
Male	115 (46.1)
Relationship status (*n* = 243)	
Married/Defacto	165 (67.9)
Single	29 (11.9)
Widowed	32 (13.1)
Divorced/Separated	17 (7.0)
Place of birth (*n* = 246)	
Australia/New Zealand	180 (73.2)
United Kingdom/Europe	49 (19.9)
Other	17 (6.9)
Source of referral to clinic (*n* = 245)	
Specialist clinician	108 (44.0)
Community palliative care service	94 (38.3)
Palliative care team on discharge from acute hospital	18 (7.3)
Palliative care team on discharge from inpatient palliative care unit	11 (4.5)
General practitioner	8 (3.3)
Other	6 (2.4)
Primary malignant diagnosis	
Gastrointestinal	80 (32.1)
Genitourinary	42 (16.9)
Lung	32 (12.9)
Breast	26 (10.4)
Gynaecology	24 (9.6)
Haematological	16 (6.4)
Skin and soft tissue	12 (4.8)
Other	17 (6.8)
Age at death, mean in years (SD)	69 (12.3)
Place of death (*n* = 204)	
Palliative care unit	112 (54.9)
Home	52 (25.5)
Acute hospital	23 (11.3)
Aged care facility	17 (8.3)
Median time between initial clinic appointment and death in days (IQR)	110 (50.8–211)
Median time between initial referral to palliative care service and death in days (IQR)	152.5 (81.8–342.5)

*SD* Standard deviation, *IQR* Inter-quartile range

^a^unless otherwise specified

### Baseline symptom intensity

**Fig. 2:**
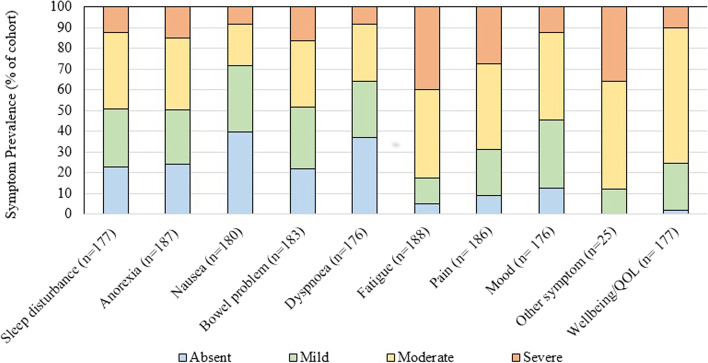
Baseline symptom prevalence and intensity

**Fig. 3:**
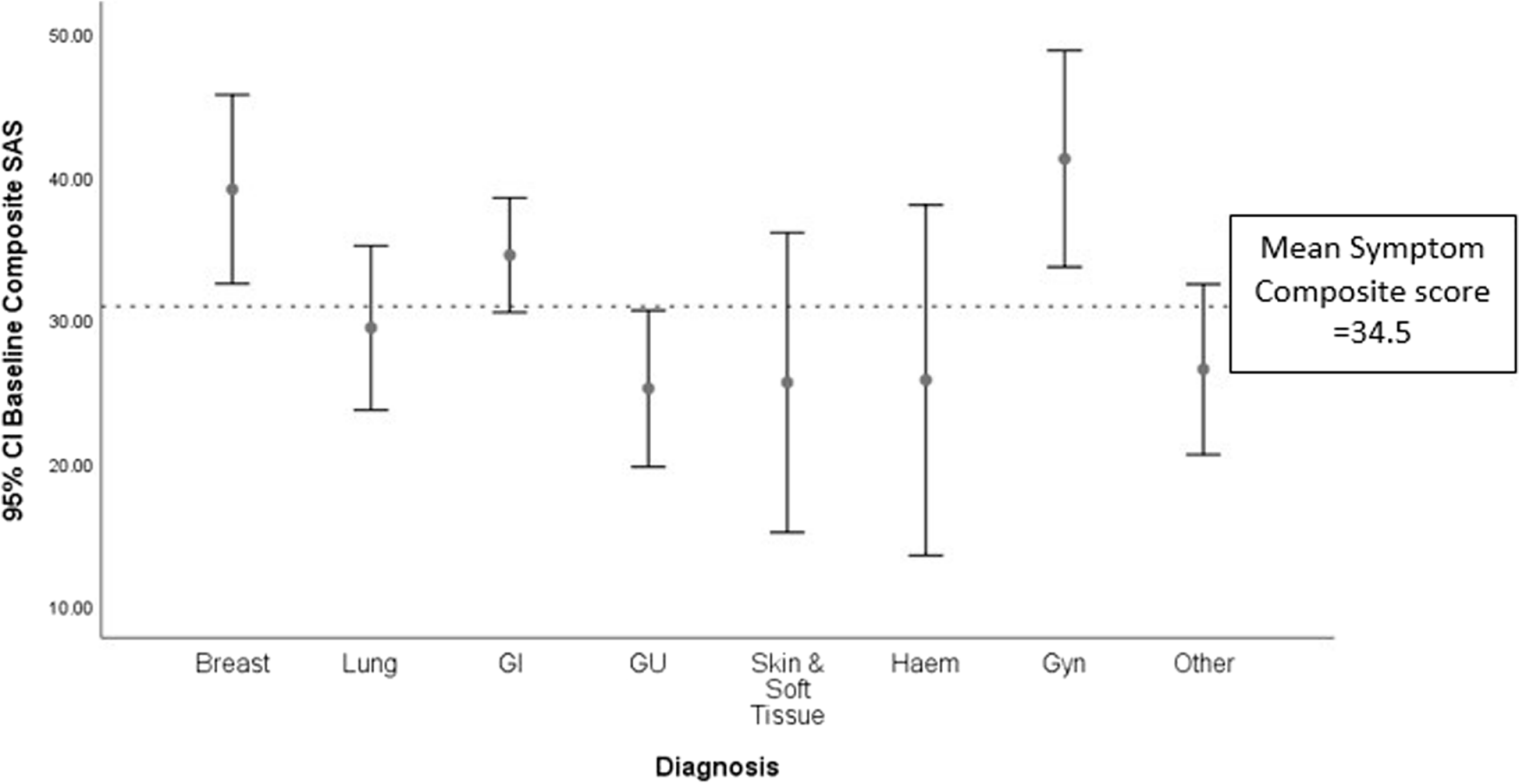
Symptom composite score stratified to primary malignant diagnosis. GI: gastrointestinal; Haem: haematological; Gyn: gynaecology

Figure 2 shows the baseline symptom prevalence categorized into absent, mild, moderate and severe subgroups. The three most common clinically significant symptoms (NRS ≥ 4) reported were fatigue (82.6%), pain (68.8%) and mood (54.5%), with close to half reporting them for anorexia (49.8%), sleep disturbance (49.1%) and bowel problems (48.6%). Three-quarters of the cohort reported clinically significant impairment in their wellbeing/QOL (75.7%). The mean baseline symptom composite score was 30.9 (SD = 15.5). Figure 3 demonstrates the mean symptom composite scores stratified to primary malignant diagnosis, with the highest scores in the gynaecology (mean 41.2, SD = 15.7), breast (mean 39.1, SD = 13.7) and gastrointestinal (mean 34.5, SD = 15.8) cancer groups.

### Symptom changes

Of the 249 patients included in our study, 126 (50.6%) attended a follow-up appointment within 8 weeks, with a median time of 4 weeks (IQR = 2.5–6.9) between clinic appointments. Table 2 summarises the change in symptom scores between the initial and follow up appointments, demonstrating a statistically significant reduction in symptom intensity for sleep disturbance (*p* < 0.001), pain (*p* = 0.002), overall wellbeing (*p* < 0.001) and symptom composite score (*p* = 0.028). There was also a statistically significant worsening in mood score (*p* = 0.002). For patients with moderate to severe symptom scores at baseline, 18–25% of patients had a clinically significant improvement (NRS ≥ 1 improvement for all SAS domains excluding pain, for pain NRS ≥ 2 improvement) in their symptom intensity between the initial and follow-up clinic appointments. However, 18–67% of patients with moderate or severe symptoms at baseline continued reporting clinically significant symptoms (NRS ≥ 4) at follow-up (Table 2).

**Table 2:** Change in SAS score

	Baseline SAS Score		Follow-up SAS score		*p*-value	Baseline NRS ≥ 4		
	*n* (%)	Median (IQR)	*n* (%)	Median (IQR)		*n* (%)	n (%) with NRS ≥ 1-point improvement	n (%) with NRS ≥ 4 at first follow-up
Sleep disturbance	177 (71.1)	3 (1,6)	76 (30.5)	2 (0,5)	< 0.001	84 (49.1)	21 (24.1)	18 (39.1)
Anorexia	187 (75.1)	3 (1,7)	74 (29.7)	2 (0,5)	0.720	93 (49.8)	22 (21.5)	16 (32.7)
Nausea	180 (72.3)	1 (0,5)	75 (30.1)	1 (0,3)	0.137	51 (28.3)	14 (27.5)	7 (30.4)
Bowel	183 (73.5)	3 (1,7)	75 (30.1)	3 (1,5)	0.062	89 (48.6)	34 (21.9)	25 (53.1)
Dyspnoea	176 (70.7)	2 (0,5)	75 (30.1)	2 (0,4)	0.580	63 (35.8)	14 (22.2)	11 (44.0)
Fatigue	188 (75.5)	7 (5,8)	75 (30.1)	3 (1,5)	0.059	155 (82.6)	34 (21.9)	53 (66.3)
Pain^a^	186 (74.7)	6 (3,8)	76 (30.5)	2 (0,4)	0.002	128 (68.8)	28 (21.9)	42 (52.5)
Mood	176 (70.1)	4 (1,6)	71 (28.5)	6 (4,7)	0.002	96 (54.5)	26 (27.1)	26 (48.1)
Other	25 (10.1)	7 (6,9)	12 (4.8)	5 (1,7)	0.021	22 (88)	4 (18.2)	2 (18.2)
Wellbeing/QOL	177 (71.1)	5 (4,7)	72 (28.9)	2 (0,5)	< 0.001	134 (75.7)	356 (26.1)	49 (63.6)
Symptom composite score, mean (SD)	183 (73.5)	30.9 (15.5)	75 (30.1)	25.9 (13.4)	0.028			

*QOL* Quality of life, *SD* Standard deviation

^a^For pain cut-off of NRS ≥ 2 improvement used; *p*-values derived from Wilcoxon signed rank test

### Clinical and service outcomes

Table 3 demonstrates the clinical and service outcomes from the initial clinic appointment. A third of patients had an opioid (30.9%) and/or an adjuvant analgesic (30.5%) initiated or titrated in the first appointment. Additionally, patients underwent opioid rotation (11.6%) or changes to their medications for nausea (7.6%), bowel issues (6%), insomnia (5.6%) or mood (3.2%). Up to 39.4% of patients were provided with education on pain management. The most common issues explored in the clinic were goals of care (30.9%), insight (27.7%), psychosocial/existential issues experienced by patients and caregivers (27.3%) and future treatment options (26.1%). After the initial clinic appointment, a quarter of the study cohort (23.3%) required an urgent admission to a palliative care unit due to inadequately managed physical or psychosocial symptoms.

**Table 3:** Clinical and Service Outcomes

	*n* (%)
Medication changes	
Opioid initiation/titration	77 (30.9)
Adjuvant analgesic initiation/titration	76 (30.5)
Opioid conversion/rotation	29 (11.6)
Anti-emetic initiation/titration/conversion	19 (7.6)
Changes to bowel regimen	15 (6.0)
Medication Management of insomnia	14 (5.6)
Commencement/Changes of anxiolytic/anti-depressant	8 (3.2)
Other medication change^a^	37 (14.9)
Referrals to	
Allied health/ancillary support	29 (11.6)
Interventional radiology and radiological investigations	24 (9.6)
Psychiatry/psychological services	18 (7.2)
Oncology/haematology/radiation oncology	9 (3.6)
Other medical or surgical	16 (6.4)
Symptom education	
Pain management	98 (39.4)
Gastrointestinal symptom and bowel management	22 (8.8)
Fatigue management	18 (7.2)
Dyspnoea management	11 (4.4)
Diet and nutrition management	10 (4.0)
Mood and insomnia management	13 (5.2)
Other symptom management	16 (6.4)
Issues explored/discussed	
Establishing goals of care	77 (30.9)
Insight	69 (27.7)
Patient and caregiver psychosocial/existential distress	68 (27.3)
Future treatment options	65 (26.1)
Prognosis	53 (21.3)
Preferred place of death	34 (13.7)
Other^b^	22 (8.8)
Disposition from initial clinic appointment	
Admit to palliative care unit	58 (23.3)
New referral to community palliative care	34 (13.7)

^a^including diuretics, mouth care, eye drops, pancreatic supplements

^b^including voluntary assisted dying enquiries, health literacy and introduction to palliative care

## Discussion

This is the first Australian study to examine the prevalence of symptoms and the impact of a supportive care clinic on patient-reported and service outcomes in cancer patients. Our findings confirm the high symptom burden experienced in this cohort, with the supportive care clinic a catalyst for admission for a quarter of the patients reviewed. We were able to affirm the benefit of ambulatory interventions by significant improvements in sleep, pain, wellbeing and overall symptom composite scores on follow-up. This study adds to the growing body of literature demonstrating the need for and efficacy of ambulatory palliative care [4, 5, 11, 20]. A direct comparison, however, of our study to other studies is challenging due to the heterogeneity in the palliative care service model globally, time to follow-up and differences in outcome measures. Whilst the SAS, which is widely used in an Australian palliative care context, and other symptom assessment tools, including the ESAS, rely on patient reporting of intensity on an NRS for a number of overlapping symptom domains, the relationship and congruence of these tools is unknown.

The high burden of clinically significant symptoms at baseline mirrors that shown in a previous retrospective study by Kang et al. [4]; however, we demonstrated improvement in symptoms occurred regardless of baseline symptom intensity. Whilst earlier studies in the ambulatory palliative care setting have shown a trend for mild baseline symptoms to intensify [4, 5], this was not evident in our study apart from worsening mood scores. Limited improvement in the domains of anorexia, nausea, bowels, dyspnoea and fatigue may be partly attributed to a smaller cohort size that prevented subgroup analysis for those with baseline moderate to severe symptoms. Additionally, symptom education was disproportionate in the initial clinic appointment, with 39.4% of patients receiving education on pain management, compared to 7.2% on fatigue and 4.4% on both dyspnoea and bowel management.

The American Society of Clinical Oncology Clinical Practice Guidelines for Palliative Care recommend that beyond symptom management, palliative care teams should assist with care coordination and explore insight, clarify treatment goals, assist with coping and provide education about illness and prognosis to cancer patients and their caregivers [26]. Whilst our SCC included aspects of this recommended practice, with up to 30% of patients having insight explored, appointment length related time constraints prevented a more comprehensive approach. As time has been described as a significant factor towards the success of palliative care, we support the recommendation for an increased patient-contact time by an extra hour a month for subsequent follow-up appointments via a face-to-face or telehealth consultation [26]. This will enable a focus on improving patient education as an essential component of the ambulatory palliative care service [27].

There are two further findings of note. Compared to previously published Australian data suggesting that cancer patients have accessed specialist palliative care for a median of 30 days (IQR 10–81 days) and community palliative care services for 62 days (IQR 26–137) before death [28], our study demonstrated that patients in our cohort accessed services for longer periods (median of 152.5 days, IQR 81.8–342.5). Though the median time from first referral to the integrated palliative care service to initial SCC appointment was 110 days, in the interim, patients would have accessed the service through contact with the inpatient consultation team or community nursing team. This reflects the transformation of the palliative care service over several years to one of early integration [29], allowing for earlier targeting of symptoms and provision of support. Secondly, using the symptom composite score as a surrogate marker for overall symptom burden, gynaecological cancer patients had the highest symptom composite scores despite representing less than 10% of the cohort. This finding is consistent with studies showing high symptom burden in this group regardless of stage and site of cancer, with younger age, ongoing treatment and history of chronic pain, depression or anxiety being predictors [30]. Likewise, patients with breast cancer, who reported the second highest symptom composite score in our cohort, are more likely to report higher levels of symptom burden with lower coping capacity and emotional distress [31]. These factors support recommendations of routine screening of targeted patients occurring concurrently with referrals based on clinician discretion [14] and may help to identify areas for future study in our institution’s cancer population who are currently not being seen in the SCC.

Our study had several limitations. Firstly, our study cohort was subject to bias. There was a high prevalence of gastrointestinal cancers in our cohort, encompassing colorectal and pancreatic cancer, and a low prevalence of skin cancers, likely secondary to our institution’s reputation as a leading colorectal surgery centre which contributed to selection bias. Additionally, of the 1060 patients seen by the service in 5 years, only 249 patients met the eligibility criteria and were evaluated, adding a further bias to the study. Secondly, given the integrated palliative care service model, which encompasses inpatient, community, consult and ambulatory services, our patients may have had access to symptom management and interventions between clinic consultations via the community and acute sector consultation and inpatient palliative care teams. Thus, it is difficult to directly attribute the changes in patient-reported outcomes to the initial SCC appointment. Thirdly, as only half of our initial cohort completed follow up SAS scores, the change in patient-report symptom scores may be underrepresented. This may explain the large proportion of patients (18–66.6%) who had moderate to severe symptoms at baseline that continued to have moderate to severe symptoms at follow-up. Finally, our follow-up time between appointments of 4 weeks is longer than the 15–21 days seen in earlier studies [4, 5, 20]. We hypothesise that this prolonged time to follow-up, due to natural progression and clinical deterioration in advanced malignancy, may have contributed to the high proportion of our patients who still had clinically significant symptoms, particularly fatigue, at follow-up.

## Conclusion

In summary, this study highlights the high burden of clinically significant symptoms and the need for effective symptom management programs in the ambulatory setting for patients with cancer. It also demonstrates the opportunity such services provide to enable patient education, psychosocial care and future planning. Considering the totality of care required across the physical, social and psycho-existential domains, more comprehensive evaluation methods to ascertain the benefits of ambulatory palliative care are needed. The results of this study will inform the development of a phase II study examining the feasibility and acceptability amongst patients and clinicians on the use of electronic capturing of more comprehensive patient-reported outcomes in the ambulatory cancer setting.

## Data Availability

The datasets generated and/or analysed during the current study are not publicly available due to organizational requirements for release but are available from the corresponding author on reasonable request.

## Abbreviations

ESAS: Edmonton Symptom Assessment Scale
NRS: Numerical Rating Scale
QOL: Quality Of Life
SAS: Symptom Assessment Scale
SCC: Supportive Care Clinic
